# Tailoring the Coefficient of Friction by Direct Laser Writing Surface Texturing

**DOI:** 10.3390/mi15010007

**Published:** 2023-12-20

**Authors:** Caterina Gaudiuso, Annalisa Volpe, Francesco Paolo Mezzapesa, Carmine Putignano, Antonio Ancona

**Affiliations:** 1 CNR IFN Institute for Photonics and Nanotechnologies, Via Amendola 173, 70126 Bari, Italy; annalisa.volpe@poliba.it (A.V.); francescopaolo.mezzapesa@cnr.it (F.P.M.); antonio.ancona@uniba.it (A.A.); 2 Dipartimento Interateneo di Fisica, Università degli Studi di Bari e Politecnico di Bari, Via Amendola 173, 70126 Bari, Italy; carmine.putignano@poliba.it; 3 Department of Mechanics, Mathematics and Management, Politecnico di Bari, Viale Japigia 182, 70126 Bari, Italy

**Keywords:** laser surface texturing, direct laser writing, friction reduction, ultrafast lasers, conformal and non-conformal contacts

## Abstract

The modification of the surface topography at the micro- and nanoscale is a widely established as one of the best ways to engineering the surface of materials, to improve the tribological performances of materials in terms of load capacity and friction. The present paper reviews the state of the art on laser surface texturing by exploiting the technique of direct laser writing for tailoring the coefficient of friction, highlighting the effect of the textures’ arrangement on the lubricated conformal and non-conformal contact behavior.

## 1. Introduction

Controlling friction is essential in many applications of everyday life. Tires, brakes, and frictional power transmission systems need high friction to function optimally. On the other hand, minimizing friction is fundamental to reduce the energy losses and improve the efficiency of mechanical systems or to diminish the wear of components and prolong their lifetime [1].

Over the past decades strong efforts have been devoted to developing strategies to manage friction, i.e., utilization of lubricants and/or functionalization of one of the surfaces of the tribo-pair [2]. Among the several solutions proposed, surface texturing has emerged as a very promising technique to control friction. It consists of fabricating on the surface of the materials a pattern of micrometric or nanometric scale defects, such as, e.g., dimples or grooves, which causes a change in the surface topography and, consequently, in the tribological behavior [3,4].

Several microfabrication technologies have been proposed for surface texturing such as mechanical micromilling [5,6], vibro-rolling [7], and reactive ion etching [8]. However, laser technology has shown noticeable advantages compared to other techniques [9,10,11,12]. The excellent laser beam quality allows tight focusing of the optical radiation into spots of a few micrometers in size, thus enabling surface modification with micrometric precision. Furthermore, it is a non-contact and extremely flexible method that allows producing large area surface patterns with different geometries by finely adjusting the laser parameters and the beam steering. When ultrashort laser pulses are used, i.e., pulse durations in the picosecond or femtosecond range, the collateral damage (i.e., burrs, recast layers, spatters or microcracks) to the surroundings of the ablated structures is negligible, thus ensuring high quality and reproducibility of the micrometric patterns [13]. Moreover, the recent development of novel temporal laser beam shaping irradiation modes, i.e., using GHz and THz bursts of pulses [14,15,16,17], promises to ensure an even better mitigation of the heat generated during the laser irradiation [18], thus resulting in significant improvements of the micromachining process in terms of ablation efficiency [15,19] and quality [20]. It was also suggested that further improvement of the ablation quality can be obtained by underwater processing of samples [21] thanks to a better management of the thermal effects. The possibility to further increase the processing speed and throughput was recently reported by Schille et al. [22], who utilized a polygonal scanner, with maximum linear beam steering speed of 950 m/s, to fully exploit the extremely high laser pulse repetition rates (up to tens of MHz) and the laser manufactures efforts to further increase it [23] to achieve surface texturing speeds up to 3.8 m^2^/min, thus paving the way for industrial exploitation of this technology.

In this review, the Direct Laser Writing (DLW) technique is described as a method to modify and control very precisely the topography of materials’ surface. Subsequently, the most commonly used tribological characterization methods are introduced. The difference between conformal and non-conformal contact is explicitly mentioned to highlight the peculiarities of these two diverse regimes. In particular, the tribological performances of several textures in a wide range of lubrication regimes are illustrated and compared with reference untextured samples.

## 2. Lubrication Regimes and Tribological Characteristics of Microstructured Surfaces

### 2.1. Stribeck Curve for Fluid Lubrication Description

**Figure 1:**
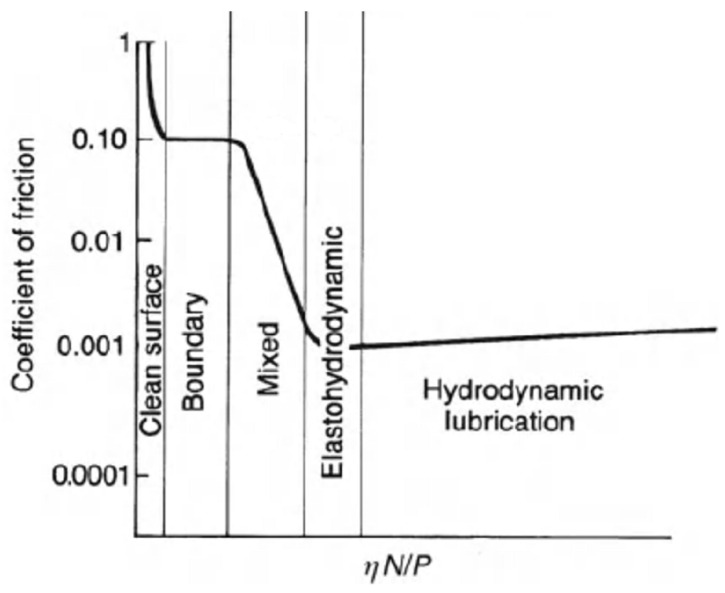
Stribeck curve. All the lubrication regimes obtainable in fluid lubrication (without external pumping of lubricant) are highlighted. Here, η is the lubricant viscosity, N is the rotational speed and P is the load. Adapted from [24], with permission from John Wiley and Sons.

Generally, the mutual motion between two solid surfaces is characterized by a quite high coefficient of friction. Therefore, lubricants, mostly liquids, are usually used to mediate the contact between the surfaces and reduce the friction, by filling the surface asperities and making them much smoother. This lubricant layer can be ensured by an external pumping mechanism (hydrostatic lubrication) or by its self-acting motion when interposed between the surfaces (fluid lubrication). The latter case is usually described by the Stribeck curve exemplary shown in Figure 1.

In Figure 1, the trend of the coefficient of friction is presented as a function of a parameter which depends on the liquid lubricant viscosity, the surface-relative speed and the load [25,26,27,28]. The exemplary curve in Figure 1 clearly shows a minimum which indicates that several mechanisms are involved in the fluid lubricant-mediated interaction between surfaces, which correspond to different lubrication regimes.

At low speed and or low viscosity, a boundary lubrication regime is found, where the coefficient of friction, though lower than in dry contact, is still high, with typical values around 0.1 or even higher [29]. In this case, a monomolecular or multimolecular lubricant layer occurs between the surfaces, whose interaction is then dominated by the contact between their asperities, as shown in [24]

A clear decreasing trend can be observed at higher speed or higher viscosity, which corresponds to the mixed lubrication regime. In this case, though direct contact still occurs, most of the bearing is supported by a lubrication film.

When high speed and high viscosity lubricants are used, hydrodynamic (HD) lubrication regime occurs. In this case, the lubricant film is much thicker (5–500 µm) than the asperities heights. Therefore, direct contact between the interacting surfaces is prevented and the coefficient of friction is significantly lower than in the two previous regimes (around 0.001).

In this lubrication regime, the formation of a hydrodynamic pressure originates from the relative motion of the surfaces, which causes a shear stress on the fluid, thus drawing it in the region between. When two parallel plane surfaces are in relative motion, a uniform fluid velocity variation from 0 (at the bottom surface at rest) to u_a_ (at the top surface) is assumed (Couette flow), with a consequent pressure gradient equal to zero [24]. This means that the pressure between the two interacting surfaces is constant (and equal to the external pressure) and this results in no load-carrying capacity obtainable in this configuration (if an external load is applied normally to the surface, the lubricant film collapses and a contact between the surfaces occurs). 

**Figure 2:**
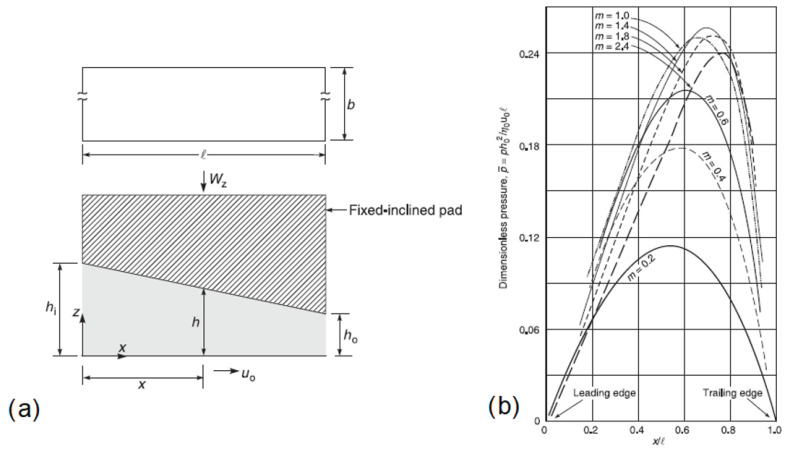
(**a**) Sketch of two non-parallel plane surfaces and (**b**) pressure build-up profile vs. the position along horizontal surface for different slopes (m). Adapted from [24], with permission from John Wiley and Sons.

However, when non-parallel plane surfaces are in relative motion, regions where a pressure build-up is experienced (as a consequence of the flow continuity) are observed. In particular, such pressure build-up regions are obtainable whenever a convergent wedge with respect to the direction of the motion is present, that is, as the thickness of the lubricant film decreases with respect to the direction of the motion. Moreover, the pressure build-up presents a maximum depending on the position along the wedge generated by the two non-parallel surfaces in Figure 2.

This implies that whenever a convergent (in the direction of the motion) wedge-shaped lubricant gap is created between two interacting surfaces, a load-carrying capacity is established [24,25]. When elastic deformations of the two interacting solid surfaces occur, elastohydrodynamic lubrication regime EHD is set, that is, a special subclass of HD. In this case, because of the elastic deformations, the lubrication layer thickness is smaller than in HD. While some asperities can still be in contact, the load is mainly supported by the lubricant film [29].

### 2.2. Controlling of Friction Properties through Surface Microstructuring

Since 1966, when Hamilton firstly proposed the possibility of modifying the surface morphology to control the tribological properties of interacting surfaces [3], surface texturing, i.e., controlled modification of the surface morphology through the introduction of micro-asperities or micro-depressions (dimples), was optimized to highlight the influence of the surface topography especially on the coefficient of friction. To this aim, several geometrical aspects were investigated, such as the micro-structures aspect ratio [30], shape [31,32,33], area density (or, equally, void ratio) [28,34,35], and the textured portion (partial or full texturing) [36,37,38]. A significant reduction in the coefficient of friction was reported, for example, by introducing a regular patter of hemispherical dimples, compared to the untextured surface case [11,39,40]. Nevertheless, it is worth mentioning that depending on the lubrication regime in the Stribeck curve, different mechanisms can be taken into account to explain the friction behavior of the textured surfaces [28].

**Figure 3:**
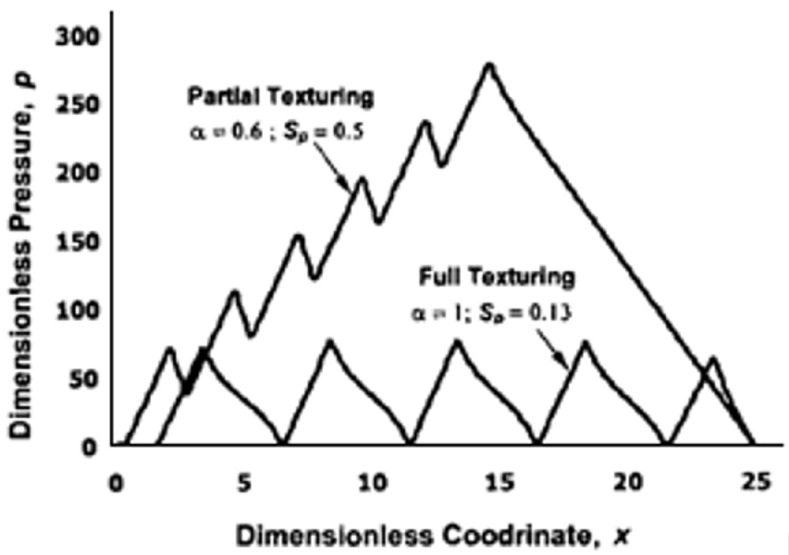
Pressure build-up in case of full texturing and partial texturing. Reprinted from [42], with permission from Taylor&Francis Group.

When low sliding speed and/or low lubricant viscosity are considered, i.e., in the boundary and mixed regime, the micro-depressions fabricated on the surface can lead to significative friction reduction by acting as lubricant micro-reservoir and as micro-traps for wear debris, thus smoothing the contact within the tribo-pair [41]. On the other hand, in hydrodynamic regime, different effects can arise. It was found that protruding asperities or depressed micro-structures can originate an additional load-carrying capacity mainly ascribed to cavitation (formation of empty bubbles within the fluid) occurring at the texture micro-depression locations [42], that is, regions where the pressure is locally equal to 0. Indeed, though local pressure buildups were obtainable via full surface texturing, no large load-carrying capacity could be actually developed through this kind of surface texturing strategy, as shown in Figure 3. Nevertheless, in Figure 3 it also is schematically shown that a partial texturing strategy can well reproduce the pressure build-up of non-parallel surfaces, thus generating the necessary convergent wedge for ensuring a high load-carrying capacity.

The role of cavitation on providing a lift force which contributes to increase the load-carrying capacity was clearly schematized by Hsu et al. [31]: when no cavitation occurs, a positive and negative pressure are observed when a convergent and divergent wedge are experienced, respectively, because of the presence of the dimples, with a null net pressure. However, with cavitation, the negative branch of the pressure is cut, thus giving rise to a net lift force. In addition, when the lubricant cavitates, its viscosity decreases, thus contributing to the reduction in the shear stress at the dimple location.

**Figure 4:**
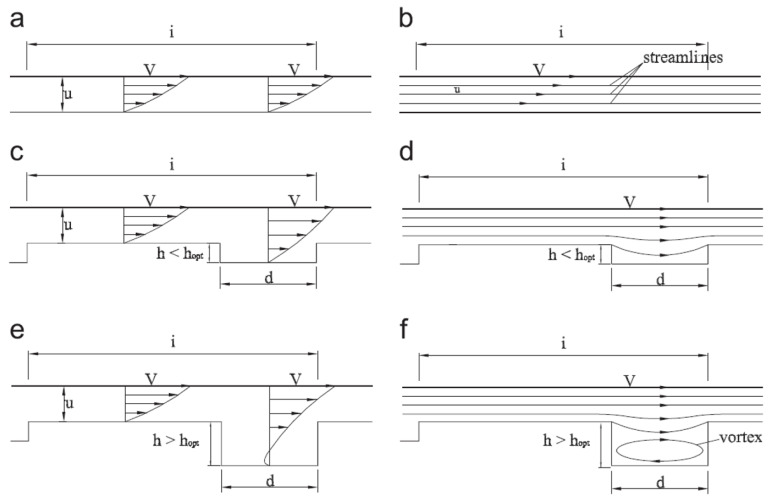
Velocity profile for (**a**,**b**) untextured case, and two textured surfaces cases: (**c**,**d**) low-depth micro-depression and (**e**,**f**) high-depth micro-depression. Reprinted from [40], with permission from Elsevier.

**Figure 5:**
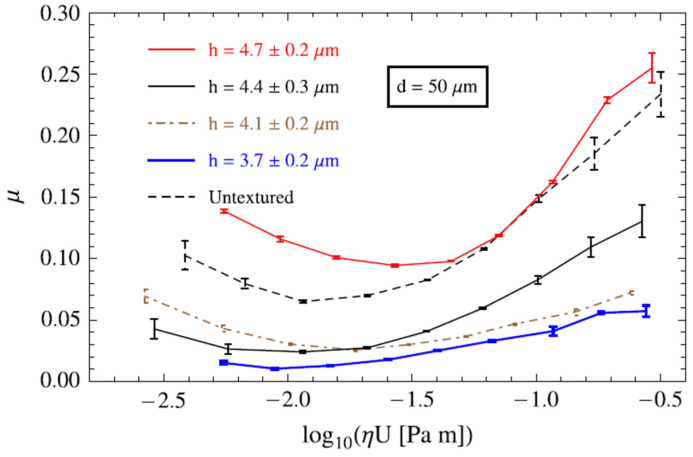
Stribeck curves for a flat test surface and for homogeneous square lattice of micro-holes having different depths. The dimples diameter and the texture area density are kept constant at 50 μm and 30%, respectively. Reprinted from [40], with permission from Elsevier.

However, if the geometrical features of the textured surfaces are not optimized, eddy-like flows (displayed in Figure 4), i.e., a vortex confined within the micro-depressions, can be generated, which cause an increase in the shear stress, dissipation of energy and, ultimately, an increase in the coefficient of friction [40] (see red curve in Figure 5).

**Figure 6:**
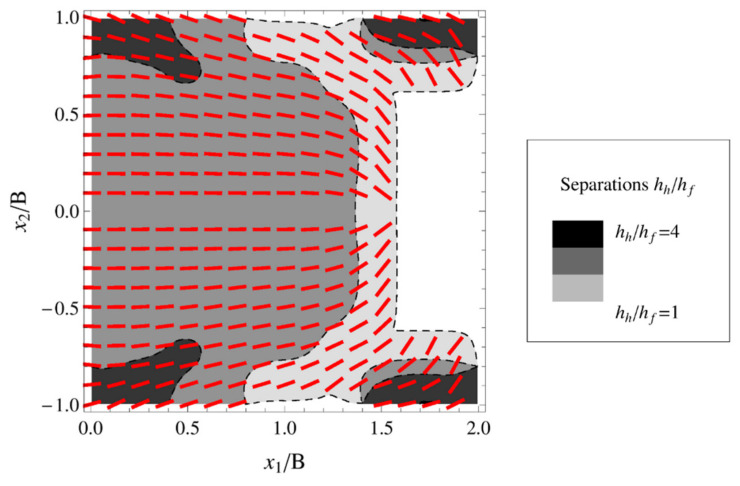
Schematics of an optimized partial textured pad for enabling synergistic interaction and load-support capability. The red stripes indicate the local inclination of the dimples. The grey scale represents the dimples’ depth. The white area corresponds to the untextured part of the pad. Reproduced from [46], under Creative Commons CC BY 4.0 license.

Though homogeneous and regular array of optimized dimples were demonstrated to be able to lead to significant friction reduction (see Figure 5), such patterns are not necessarily the best to optimize the tribological performances of the materials [36,43,44]. Theoretical investigations based on the mean field theory of texture hydrodynamics (BTH) instead revealed the existence of optimized non-homogeneous patterns able to generate collective effect of the dimples, resulting in macro-hydrodynamic friction regime during the interaction within the tribo-pair [36,43,45]. According to these studies, to enable such an effect, the micro-dimples should be inclined and misaligned with respect to the sliding direction, to channel the fluid and prevent it from escaping from the contact region. An example of such texture is presented in Figure 6.

In this way, thanks to the collective averaged effect of the dimples, the fluid is oriented within the contact domain, thus ensuring high-bearing load support and, consequently, friction reduction, creating the possibility to generate special superbearing configurations [36,43,46].

## 3. Direct Laser Writing with Ultrashort Pulses for Surface Topography Modification

As described in Section 2, the tribological behaviour of a surface is strictly related to its topography at the micro- and nano-scale. Therefore, controlling friction at the interface between two sliding surfaces requires us to properly design and precisely fabricate the geometrical features of the texture pattern. Laser surface structuring, consisting of creating a high-density matrix of such micro-structures on a solid surface by laser irradiation, meets perfectly these requirements [9,47,48]. This is due to the peculiar timescale that characterizes the laser-matter interaction: when picosecond or femtosecond pulses are exploited, the laser energy is quite instantaneously absorbed by the electron system. Being the pulse duration in ultrashort regime much shorter than the electron-phonon coupling, i.e., the time needed for the energy to be transferred from the electron system to the lattice, during the laser irradiation with picosecond and femtosecond pulses, the lattice temperature does not increase significantly. Therefore, the absorbed laser energy remains confined within the irradiated zone, and the thermal damage outside is importantly limited [13,49,50], thus minimizing the heat affected zone (HAZ). This results in a very precise and accurate structure fabrication, with almost no burrs and rims, virtually on every material, from metals to glass, also including biological samples [15,19,51,52,53]. 

Among the laser surface texturing techniques [54,55,56,57,58,59,60,61,62,63], Direct Laser Writing (DLW), based on the direct removal of material as a consequence of the laser irradiation, is one of the most exploited to generate individual micro-ablated structures with different shapes, depths and spatial arrangements [27,28,64,65]. The conventional approach for DLW surface structuring is based on the CAD/CAM concept, where the laser source is exploited as non-contact tool for surface modification. A PC-interfaced galvoscanner equipped with a F-theta lens is typically used to focus and move the laser beam on the samples’ surface [34]. Generally, the focused beam diameter results are around 10 μm or more. However, it is also possible to exploit microscope objectives for reaching much smaller focal spot sizes and PC-interfaced motorized axes to move the samples underneath the laser beam [65]. 

In this way, it is possible to micro-mill micro-structures layer after layer to finally reach the desired depth by scanning the target to be textured. When pulsed laser sources are used, the repetition rate and the scan speed (either of the galvo-scanner or of the motorized axes) are properly selected so that the number of pulses overlapping in the same spot is high enough to reduce the threshold fluence and facilitate ablation [14,66], but do not cause heat accumulation [67]. Moreover, direct laser writing is usually carried out just above the threshold fluence, to ensure high accuracy and reproducibility of the ablation process. 

**Figure 7:**
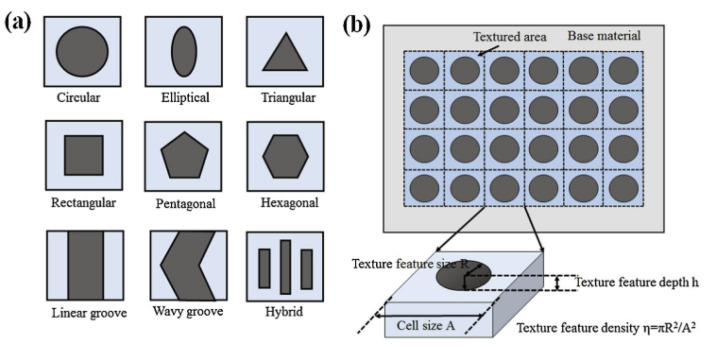
(**a**) Possible dimples shapes and (**b**) definition of the texture density (or void ratio—VR). Adapted from [64], with permission from Elsevier.

The general procedure described above allows generating several types of micro-structures, e.g., microgrooves and micro-dimples with a wide range of different geometries, as shown in Figure 7 [64]. 

**Figure 8:**
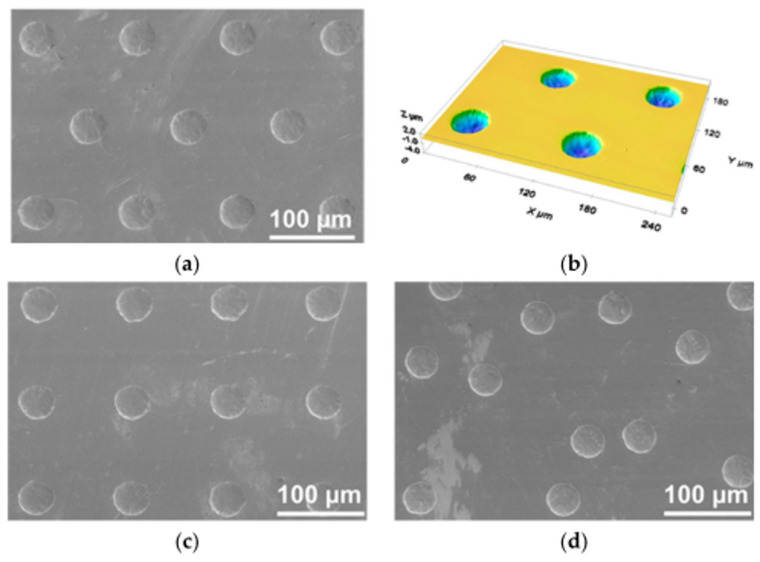
SEM and optical profilometer images of laser textured surfaces with different circular (diameter = 40 µm) dimples arrangements: (**a**,**b**) hexagonal; (**c**) grid; (**d**) random. Reproduced from [30], under Creative Commons CC BY 4.0 license.

**Figure 9:**
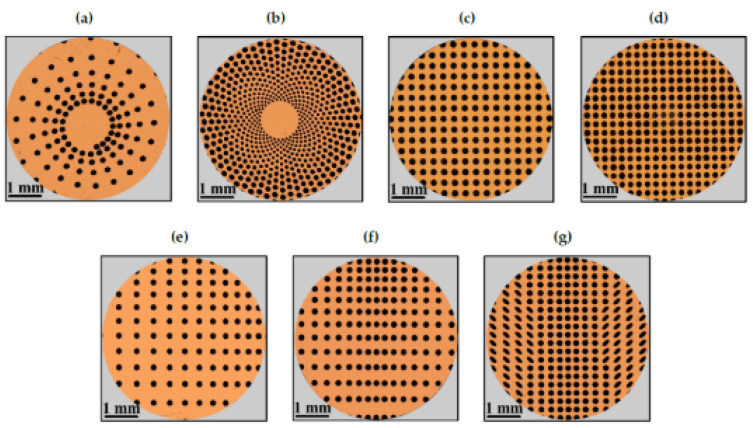
Optical microscope images of laser textured 100Cr6 steel samples. (**a**) Nautilus; (**b**) sunflower; (**c**) grid (VR = 33%); (**d**) grid (44%); (**e**) diagonal; (**f**) hydrostep; (**g**) fishbone. Reproduced from [34], under Creative Commons CC BY 4.0 license.

Thanks to the extreme flexibility of the laser technology, very complex patterns designs for tailoring the surface tribological properties can be fabricated, e.g., (1) homogeneous textures to highlight the influence of the geometry and the density distribution (or void ratio VR) of the textured features (see Figure 8a,c and Figure 9a–d) on the frictional behavior, over the whole Stribeck curve and (2) asymmetric, non-uniform and/or partial textured directional designs, to exploit the morphological anisotropies to obtain equivalent convergent or divergent wedges, depending on the direction of the flow (see Figure 8d and Figure 9e–g), to generate equivalent convergent wedges to induce high load-carrying capacity especially in the HD regime, as described in Section 2. 

## 4. Laser Surface Texturing for Friction Reduction

As described in Section 2, different mechanisms are responsible for the frictional properties of textured mating surfaces. Therefore, finding general guidelines to design novel textures configurations capable of optimizing the tribological characteristics of surfaces in different working conditions [44,68,69,70,71,72] is not an easy task.

In the present section, we aim to unveil the different outcomes of laser surface texturing on conformal and non-conformal contacts to highlight the peculiarities of these two diverse conditions. Conformal contacts can be usually found in journal bearing mating surfaces, which fit tightly enough that the radial clearance between the bearing and the journal is around one thousandth of the journal diameter. In this case, the applied load can be supported over a large area (significantly larger than the typical size of a laser ablated feature), which also remains substantially unchanged as the load increases. Instead, non-conformal contacts can be found whenever the contact area is small, due to the mating surfaces not fitting tightly, e.g., rolling bearings, cams and gears. In this case, the load is supported over a very small contact area (comparable to the fabricated surface structures) where, consequently, stresses are very high. Though the contact area widens with the load it remains smaller than in the conformal case, with very few micro-structures involved in the mating contact.

### 4.1. Conformal Contacts

As mentioned, according to their arrangement over the surface it is possible to highlight the role of the fabricated micro-structures on the frictional behavior over the whole Stribeck curve, in terms of lubricant film increase, wear particle micro-traps, cavitation sites and pressure build-up assemblies. In the present subsection, conformal contact conditions will be taken into account by explicitly considering both homogeneous and non-uniform laser textured patterns.

#### 4.1.1. Homogeneous

**Figure 10:**
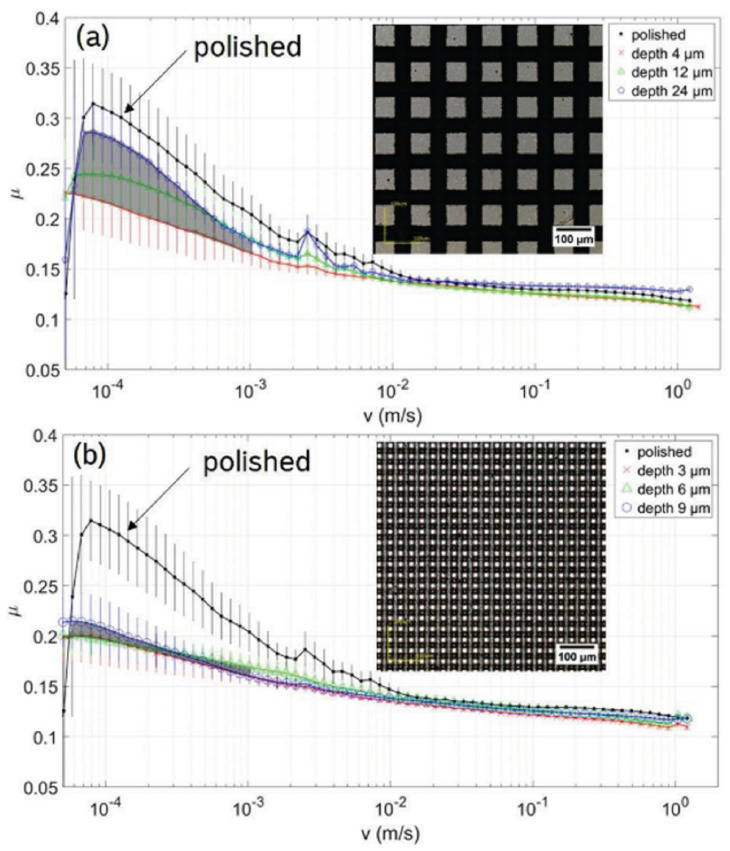
Stribeck curves for crossed grooves (**a**) period of 30 µm and (**b**) 12 µm, for different depths, obtained by DLW. The grey area indicates the COF at small sliding velocities. Reproduced from [47] with permission from Japan Laser Processing Society.

Textures consisting of uniformly arranged micro-grooves were found to produce a significant increase in the coefficient of friction (COF) in comparison with the untextured reference case [73]. This behavior was ascribed to the grooves acting as micro-ducts for the lubricant that flowed out of the contact area thus leading to starved lubrication [28]. In fact, in such open structures, i.e., channel-like features, the lubricant can be guided and transported away from the contact area, thus leading to an increase in the COF. Nonetheless, Stark et al. [47] showed that an isotropic distribution of the lubricant is obtainable with crossed groove (see Figure 10, which resulted in a lower COF especially for low sliding velocity (an overall reduction in the COF about 33%) as long as small sizes of the micro-features were exploited (width of the groove around 12 µm) (see Figure 10). 

Among the symmetric patterns obtainable by laser surface texturing through DLW, uniform grids of circular dimples are certainly the simplest and most used. Such designs were successfully performed on several materials, such as stainless steel [28,34,40,74], titanium [75], ceramics [76], rubber [77], with different dimples shapes, sizes (in terms of diameter and depth) and void ratio [78,79]. 

**Figure 11:**
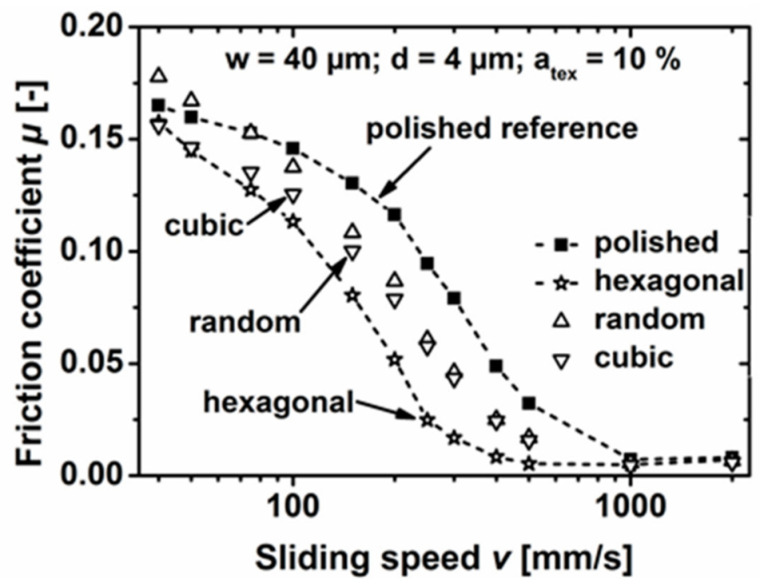
COF vs. sliding for hexagonal, cubic and random arrangement of 40 μm dimples with a depth of 4 μm, with a fixed VR = 10%. The untextured polished surface is shown for comparison. Reproduced from [30], under Creative Commons CC BY 4.0 license.

Based on the results of theoretical models developed to demonstrate the potential of surface texturing on friction reduction [12,43,45,80], it was found that an aspect ratio of the dimples (defined as the depth over the diameter) of 0.1 allowed the highest friction reduction over the whole Stribeck curve [12,80]. Such predictions were also experimentally confirmed by Schneider et al. [30], though several contradictions could be found depending on the viscosity of the lubricant used for the tests [8,81,82]. In [30], it was also shown that once setting the dimples geometrical configuration (diameter = 40 µm; depth = 4 µm; void ratio = 10%), a random distribution of the micro-features allowed a COF reduction of 48% (comparable to the uniform grip) compared to an untextured surface. Much better results could instead be obtained (COF reduction around 82%) for a uniform hexagonal distribution, as visible in Figure 11.

Beside the dimples arrangement, it was also revealed that, depending on the lubricant [83] used for the tests and the velocity gradient, i.e., the difference in sliding speed over the textured area involved in the contact [84], an optimal value of the dimples’ diameter exists [83,84,85], which allows for a reduction in the COF of up to 80%. Ancona et al. [37] showed that depending on the diameter of the dimples, also an ideal value of their depth can be found, though not fulfilling the aspect ratio of 0.1. In fact, the authors found that while for 40 µm diameter dimples the best friction performances were found for a depth around 11 µm over the entire Stribeck curve, for much bigger dimples (diameter of 188 µm), the optimal depth was around 7 µm. To explain the existence of an optimal depth over which the friction increased, the authors invoked the formation of vortex which leads to a deterioration of the frictional characteristics [40].

Another important parameter influencing the tribological performance of a textured surface is the void ratio (VR), that is, the ratio between the total dimples area and the total surface. In [30] the coefficient of friction of textured sample with VR = 5% was slightly higher than a polished one while the best results in terms of COF reduction were obtained with a VR = 10%. A further increase in the VR above 10% resulted in a progressive increase in the COF over the whole Stribeck curve, in contrast with theoretical predictions and other experimental works, where a constant reduction in the COF was reported until a saturation was reached, around 30%, likely originating from the collective operation of the texture and from the enhanced trapping capability for debris particles and lubricant [28,34,35,42,82]. The authors ascribed the better results obtained with a VR = 10% compared to VR = 5% to a collective effect behavior of the dimples (the macroscopic effect was not the sum of the outcomes of the single dimples), though omitting a comprehensive explanation of the increase in the COF at VR > 10%.

**Figure 12:**
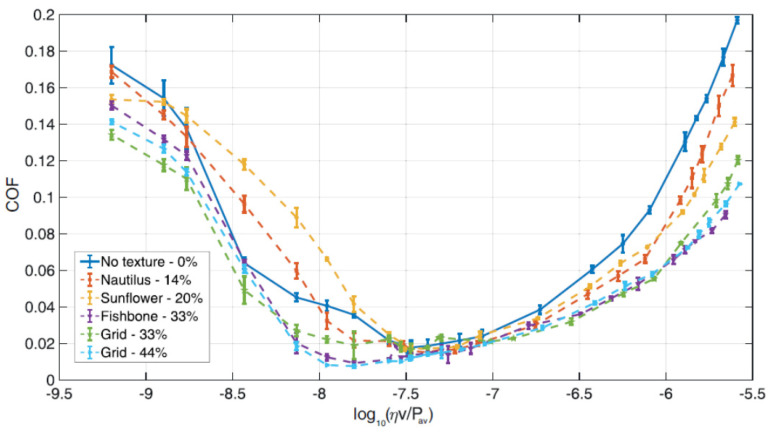
Coefficient of friction vs. Hersey number for the textures shown in Figure 9a–d,g. The results for the untextured surface are also shown. Reproduced from [34], under Creative Commons CC BY 4.0 license.

Putignano et al. focused on several symmetric patterns generated on 100Cr6 steel spherical caps (diameter~5 mm), i.e., nautilus, sunflower, fishbone and two uniform grids with different void ratios (33% and 44%) as shown in Figure 9a–d. The tribological tests performed in conformal contact conditions (Pin-on-Disk configuration) revealed that in the mixed lubrication regime, only the textures with a high void ratio (33%) allowed a reduction in the friction [34], as noticeable in Figure 12. In this case, the contribution of the dimples to the debris trapping and lubricant film preservation overcame the stress intensification possibly due to the dimples’ edges. Regarding the full hydro-dynamic lubrication regime, all the textures showed increasing beneficial effects in terms of friction reduction as the VR increased [42], ascribed to cavitation occurring at the dimples’ sites [86].

**Figure 13:**
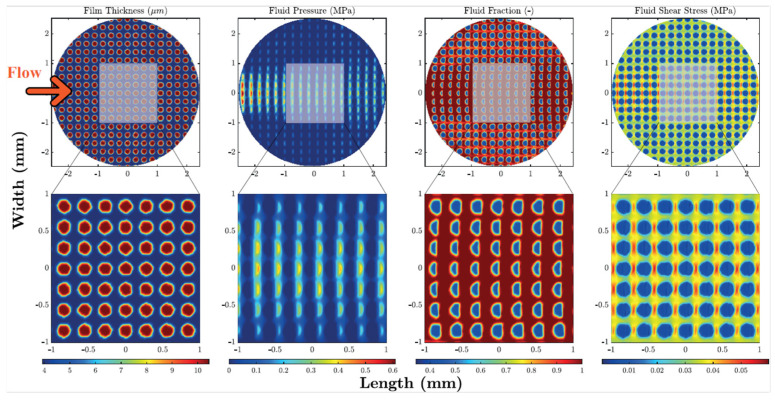
Numerical results for film thickness, fluid pressure, fluid fraction and average shear stress for the uniform grid (VR = 44%) and Hersey parameter (H = η*v/P, where η is the dynamic viscosity, v is the sliding speed and P is the normal load per length of the contact) equal to H = 2.35 × 106. Reproduced from [34], under Creative Commons CC BY 4.0 license.

Consequently, the authors argued that while the main parameter responsible for the friction reduction was the void ratio, the specific arrangement of the dimples on the surface played a negligible role. Numerical outcomes, as reported in Figure 13, showed that the main origin of the friction decrease is related to cavitation occurring in each dimple. 

As expected, the lubricant cavitation at the divergent zone, i.e., each dimple’s inlet, resulted in a drop of the fluid pressure and in the fluid rupture. However, when in the convergent region, i.e., at each dimple’s outlet, the pressure increased, and the lubricant film restored. This finally resulted in a higher lubricant film thickness and lower effective viscosity originating from cavitation, which led to an overall decrease in the shear stress.

**Figure 14:**
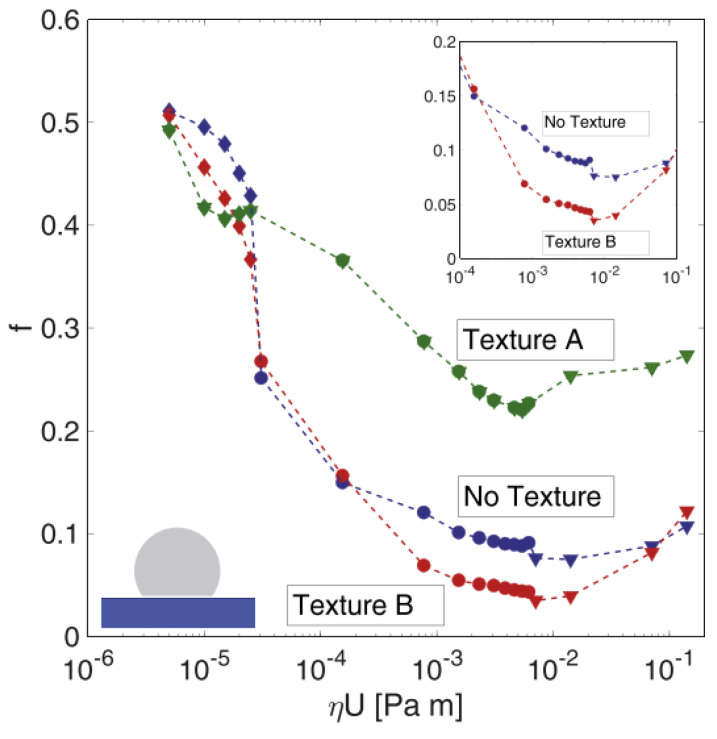
COF vs. ηU in pin-on-disk tests. The normal load was of 1 N. Reproduced from [77], with permission from Elsevier.

The same authors fabricated two uniform grids of circular dimples also on a fluoroelastomer (commercial name Viton rubber). Soft materials are likely to show a visco-elastohydrodynamic lubrication (VEHL) trend, but, even under these conditions, micro-texturing proved to be useful to decrease friction. Indeed, the two tested patterns differed for the dimensions of the dimples and their mutual distance. Texture A was fabricated with large and deep dimples (diameter of 100 µm with a center-to-center distance of 150 µm and depth of 50 µm), with the aim of maximizing the amount of lubricant retainable inside; Texture B, instead, was fabricated with much smaller and shallower dimples (diameter of 50 µm with a center-to-center distance of 100 µm and depth of 10 µm), to minimize the chance of eddy formation, which would have a detrimental effect on the COF [37]. Testing of such textures in conformal contact conditions showed a COF reduction of 60% obtainable with Texture B in the mixed lubrication regime, while a remarkable deterioration of the frictional performances was observed for Texture A (see Figure 14).

**Figure 15:**
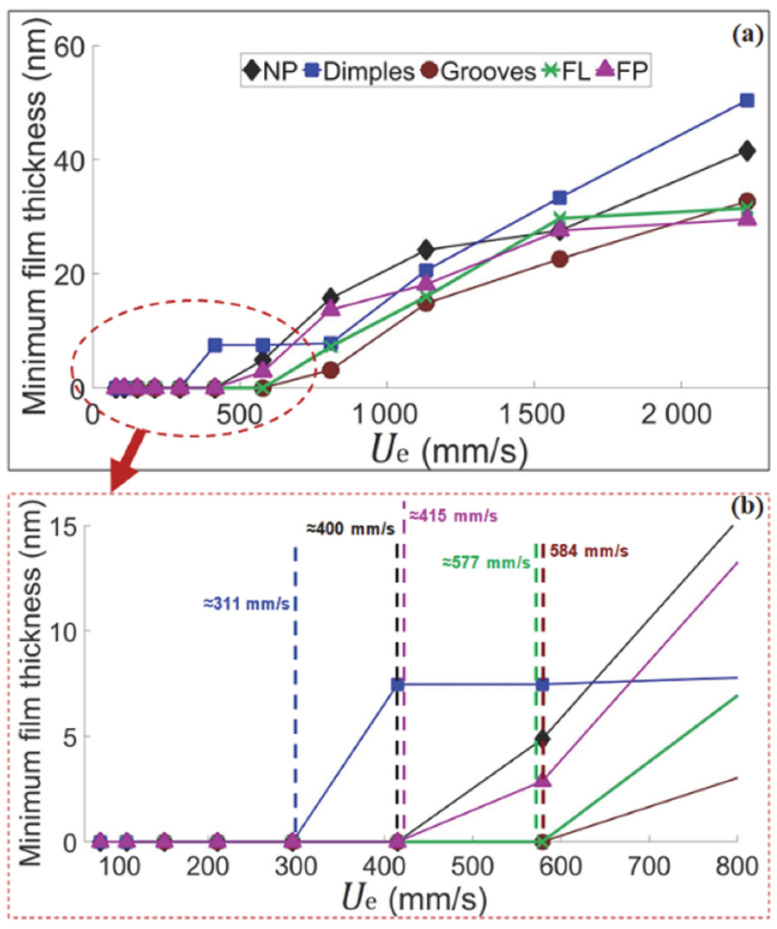
(**a**) Measured minimum film thickness for untextured (NP) and textured balls varying entrainment speed. (**b**) Details of the low-speed region (<800 mm/s) and lift-off speed values (vertical dashed lines). The blue square correspond to dimples, the claret circles to grooves ablated by DLW, the green cross to longitudinal grooves (FL) ablated by DLIP, and the purple triangles to perpendicular grooves (FP) ablated by DLIP Reprinted from [65], under Creative Commons CC BY license.

The differing behaviors of the two textures in the mixed lubrication regime was ascribed to two significant effects occurring in such testing conditions (inset Figure 15): (1) separation of the two mating surfaces due to the dimples acting as lubricant micro-reservoir; (2) cavitation inside the dimples [28,87]. Nonetheless, when the dimples are too deep, it is known that fluid turbulence is generated, thus causing severe detrimental effects of the tribological properties [37]. This is the reason why Texture A, though its dimples were able to store much more lubricant thanks to their geometrical features, presented much worse outcomes in terms of friction in the mixed and hydrodynamic lubrication regimes. Conversely, in the boundary regime, where the fluid speed was much slower, cavitation and fluid turbulence are not remarkable, while the increment of lubricant supply and the wear particles capture occurring at the dimples’ sites can explain the reduction in the COF of around 20% obtained with Texture A.

**Figure 16:**
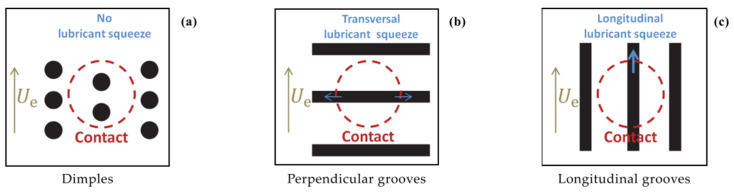
Sketch of the lubricant behavior for (**a**) dimples, (**b**) perpendicular, and (**c**) longitudinal groove textures. Reprinted from [65], under Creative Commons CC BY license.

Boidi et al. [65] recently also demonstrated the possibility to apply direct laser writing technique to curved samples. To this aim, they textured ball samples with circular dimples and grooves. The ball-on-disk tests with lubricant carried out under elasto-hydrodynamic lubricated contact and presented in Figure 15 revealed that also for curved samples, dimples were able to promote friction reduction (up to 20%), whereas higher friction was found with longitudinal grooves. These results were interpreted in terms of capacity of lubricant film retainment on the surface: in fact, while dimples were able to trap the lubricant, also thanks to their smaller dimensions compared to the contact area (see Figure 16), longitudinal grooves caused the lubricant to flow away the contact area, thus preventing the formation of local lubricant pressure, in good agreement with [28,73] (see Figure 16).

#### 4.1.2. Non-Uniform

In Section 2, it was mentioned that, even though dimples can be able to store the lubricant, regular arrangements of micro-structures fail to orient its flow because they provide isotropic conductivities and then they are not necessarily the best to reduce the coefficient of friction [36,43,44]. Conversely, theoretical studies demonstrated that non-homogeneous surface textures could be able to imitate convergent wedges, and/or control the local micro-fluid dynamics [36], thus generating superior load-carrying capacities. 

Inspired by these theoretical predictions, Ancona et al. [37] compared the outcomes of full textures (FT) consisting of circular dimples (VR = 21%, dimples diameter of 40 µm and dimples depth of 11 µm) with partial textures (PT), where only one half of the sample surface was structures. The different orientation of the partially textured samples with respect to the sliding direction defined also their potential in generating a built-up pressure and then an additional load-carrying capacity. In fact, the partially textures samples were tested in two different configurations, one with the structured area at the contact inlet, called PT and the other with the structured area at the outlet, called PT-R. Therefore, PT textures were supposed to act as convergent wedges, thus increasing the load capacity and reducing friction. Conversely, PT-R is supposed to worsen the frictional behavior, due to a pressure drop originated by an equivalent divergent wedge.

**Figure 17:**
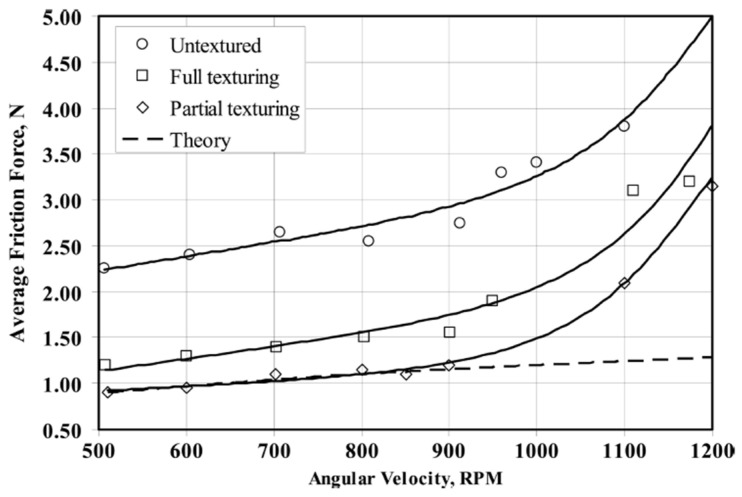
Average friction force vs. angular velocity, for an external pressure of 0.3 MPs. The dashed line represents the theoretical prediction by Kligerman et al. Reprinted from [38], with permission of Taylor&Francis Group.

The experimental results showed that full textured samples ensured 85% reduction in the COF over the whole Stribeck curve [37]. On the contrary, in contrast with the predictions [38,39], though a higher load capacity was expected (equivalent convergent wedge) [36], PT generally led to an increase in the friction. The authors argued that the reduction in shear stress at the dimples’ sites leading to the increase in the lubricant thickness [81] obtained with full texturing was much more significant than the load-bearing capacity of partial texturing. Different results were found by Ryk et al. [38] who reported a significant friction reduction with partial laser surface texturing (see Figure 17).

Here, partial texturing clearly acted as a converging wedge by generating collective effects of the dimples and thus leading to a further friction reduction (compared to full texturing) of around 29%.

Another important aspect to be considered when designing a surface for tribological applications is the mutual orientation between the dimples and the sliding velocity. This was investigated in [37] taking into account direction of the major axis of elliptical dimples with respect to sliding velocity and analyzing how the COF was influenced. The highest friction reduction was reported when the sliding direction was perpendicular to the major axis of the ellipses, at an angle of 90°. Interestingly, at an inclination angle of 0°, an increase in friction was found compared with the untextured surface. These results, in good agreement with [31] and the theoretical predictions [32], suggested that rotating the lubricant flow would result in a switch of the frictional properties (high or low friction when the major axis of the ellipses is parallel or perpendicular to the lubricant flow, respectively). This opened to the possibility to dynamically adjust the same textured component to operate in different working conditions. 

**Figure 18:**
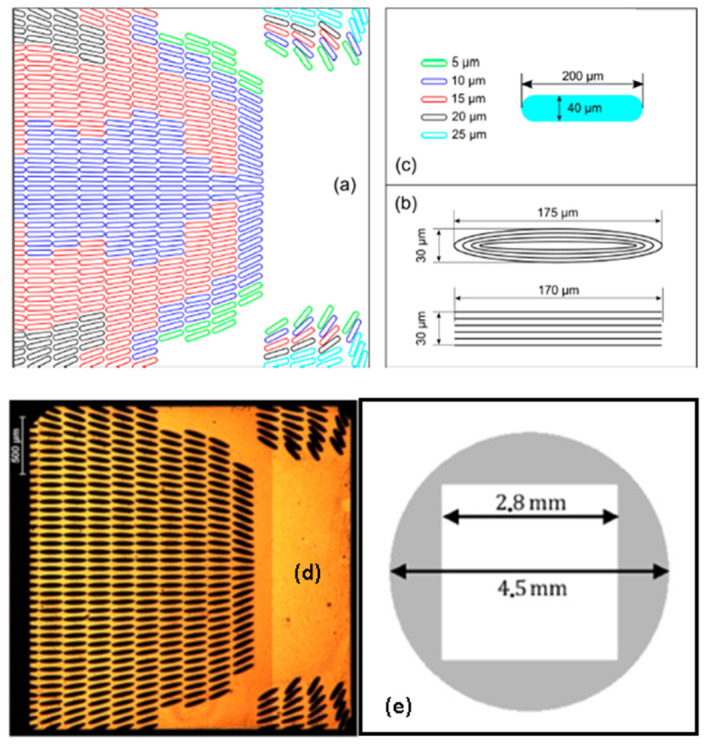
(**a**) Schematic of the texture pattern; (**b**) ablation strategy to realize elliptical and rectangular dimple; (**c**) dimples size; (**d**) sketch of the truncated bearing sphere where the grey area was removed by laser milling for a depth of approximately 100 µm; (**e**) optical microscope image of the squared pad textured with elliptical dimples. Reproduced from [46], under Creative Commons CC BY 4.0 license.

This is the reason why a partial texture characterized by a lattice of finite-sized grooves having a certain local angular misalignment with respect to the sliding direction was also proposed. The texture geometry was designed for a square pad and optimized by adopting a multigrid procedure based on a genetic algorithm. A schematic representation of the texture pattern is shown in Figure 18a. It consisted of elongated dimples partially covering the pad area. The color scale indicates the different depth of the dimples. They were distributed and aligned in such a way to hinder the flow of the lubricant towards the lateral direction, thus preventing any immediate leakage and, hence, friction increase. 100Cr6 steel samples (truncated spheres) were used for the fabrication of two prototypes of non-uniform textures for super bearing effect generation. Two different textures were performed, i.e., consisting of elliptical and rectangular dimples. Such textures were generated within squared pads (side = 2.8 mm) obtained by laser micro-milling of the area outside the square. The milling depth was around 100 µm, one order of magnitude higher than the expected average fluid thickness under the bearing. A flat untextured control pad was also fabricated for reference measurements. 

Thanks to their inclination and the micro-herringbone construction, the dimples close to the lateral boundaries enabled redirecting the fluid towards the internal portion of the contact. This determined many fluid particles sheared at the contact interface and, consequently, an increase in the bearing pressure. Groups of dimples on the outlet corners of the pad had been designed with the aim of lengthening the fluid path under the pad domain. Indeed, they should have redirected part of the flow, which normally would have exited from the middle part of the rear side, towards the rear part of the pad. In this way, each fluid particle would have exerted a prolonged shearing action, thus significantly increasing the load-support capabilities of the pad. The overall void ratio of the textured area was approximately 50%. 

**Figure 19:**
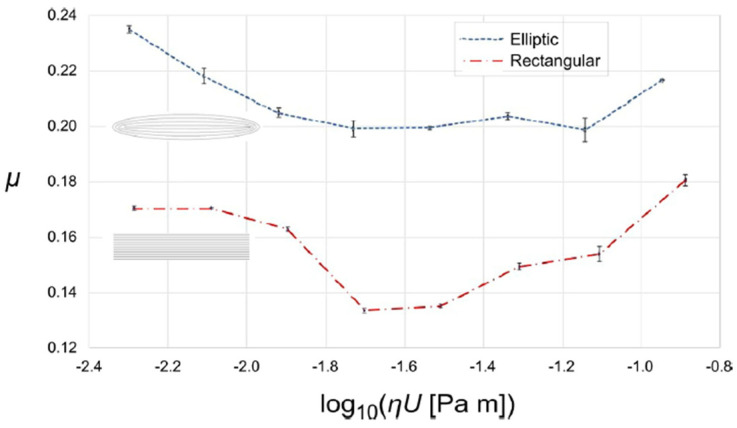
Stribeck curves of the two non-uniformly textured square pads with rectangular and elliptical dimples, with an applied normal load of 1 N. Reproduced from [46], under Creative Commons CC BY 4.0 license.

The Stribeck curves obtained by applying a load of 1 N to the two square non-uniformly textured pads (elliptical and rectangular dimples) are shown in Figure 19. For both samples, the three lubrication regimes were clearly visible. At low sliding speeds, i.e., below 0.03 m/s, the friction coefficient was relatively high, as expected in boundary lubrication. Between 0.04 m/s and 0.1 m/s, friction progressively dropped as typical of the mixed lubrication regime. The minimum friction coefficient was measured around 0.2 m/s. Then a hydrodynamic regime was established in which friction increases with the sliding speed due to the resistance of the fluid.

**Figure 20:**
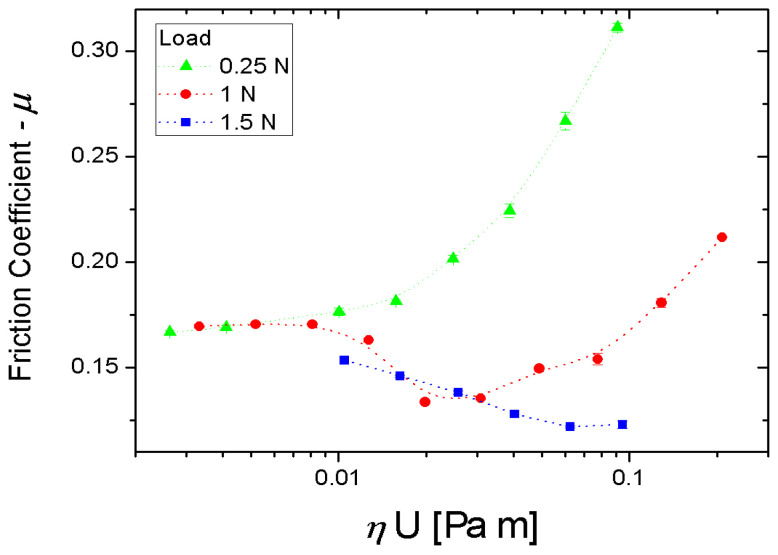
Stribeck curves of the non-uniformly textured square pads with rectangular dimples at three different normal loads of 0.25 N, 1 N and 1.5 N, respectively. Reproduced from [46], under Creative Commons CC BY 4.0 license.

The comparison between the two samples showed that the rectangular dimples had a 20% better performance in terms of friction reduction than the elliptical ones. This was ascribed to the elliptical dimples having a slightly smaller area compared to the rectangular ones. Therefore, the overall area density of the pad with rectangular texture was higher, which reduced the contact area in the boundary and mixed lubrication regimes as well as the interfacial separation in hydrodynamic lubrication conditions. The result was a lower friction of the pad with rectangular dimples. For this reason, the latter pad was selected for further tests with different loads of 0.25 N, 1.0 N and 1.5 N, respectively, and sliding speeds varying from 324 mm/s to 15 mm/s. The corresponding friction curves are plotted in Figure 20. Here, it can be noticed that for the lower load of 0.25 N a remarkable hydrodynamic behavior was established which extended over almost the entire range of investigated sliding speeds. This was a clear indication that the anisotropic texture patterns were able to successfully redirect and maintain the lubricant under the contact area. In addition, at the higher load of 1.5 N the friction coefficient significantly reduced with the sliding speed, even below the minimum value registered at 1 N. This was again ascribable to the collective flow action of the dimples that increased the load-support capabilities of the micro-structured pad.

**Figure 21:**
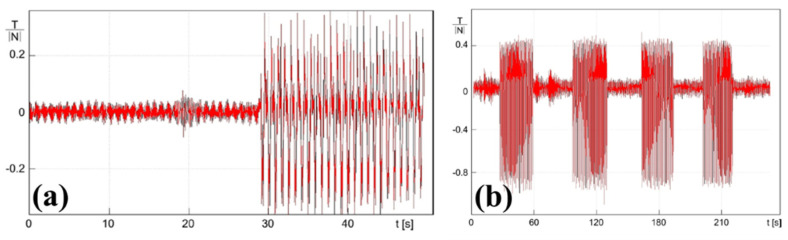
Friction coefficient behavior calculated as the tangential force T to the normal force N ratio, measured by running the tribometer in reciprocating mode with untextured (**a**) and non-homogeneously textured square pad (**b**). The linear translation speed was 136 mm/s reversed with a frequency of 1 Hz, and the applied load was 0.25 N. Reproduced from [46], under Creative Commons CC BY 4.0 license.

In order to further prove that such tribological effects observed were originated by the anisotropic texture, the authors tested the same textured sample by operating the tribometer in reciprocating mode, where the sliding speed was periodically reversed, comparing the results to the untextured case. Figure 21 shows the results obtained for the untextured sample. The graph reports the ratio between the measured (apparent) tangential force and the normal load as a function of contact time and clearly showed positive or negative oscillation (depending on the verse of the sliding direction) of equal amplitude. 

The textured sample performed differently, as shown in Figure 21b. Here, there was a substantial difference in the amplitude of the tangential force measured depending on the orientation of the texture with respect to the sliding verse. When the textured area was at the inlet, the tangential force was half the value measured in the opposite direction. Therefore, the non-uniform micro-texture largely affected the friction characteristics of the contact, in agreement with the fluid-dynamic predictions. In Figure 21b, the same measurement was repeated for four different time intervals exhibiting high reproducibility and proving the micro-hydrodynamic effect of the anisotropic surface texture.

**Figure 22:**
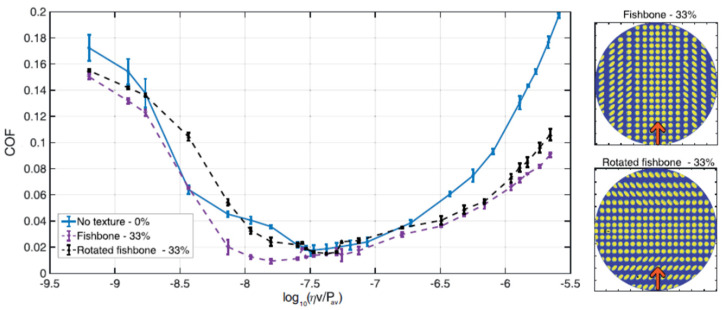
Coefficient of friction (COF) vs. Hersey number log10(ηv/Pav) for the Fishbone and the rotated Fishbone textures (see the inset on the right). The load is constant and equal to 1 N. Untextured surface is also shown for a comparison. Reproduced from [34], under Creative Commons CC BY 4.0 license.

The importance of the flow direction with respect to the asymmetric texture arrangements was also demonstrated in [34], where the authors analyzed the outcomes of three asymmetric patterns, i.e., fishbone, diagonal and hydro-step, as in Figure 9e–g, in terms of friction reduction. Rotated fishbone pattern led to different outcomes if compared to the conventional fishbone (see inset in Figure 22) due to the lubricant flow initially directed over the ellipses. This affected the micro-cavitation inside the dimples and caused a friction increase [88].

Furthermore, using directional diagonal and/or hydro-step patterns, equivalent wedges (convergent or divergent) could be created. This is what was experienced with the diagonal and the hydro-step textures, where besides the local effects due to the presence of the dimples, some macroscopic and collective effects were also observed. An equivalent convergent wedge was expected to offer a better hydro-dynamic support, ensured by a thicker minimum oil film, thus resulting in a lower friction [26,27,89,90]. 

**Figure 23:**
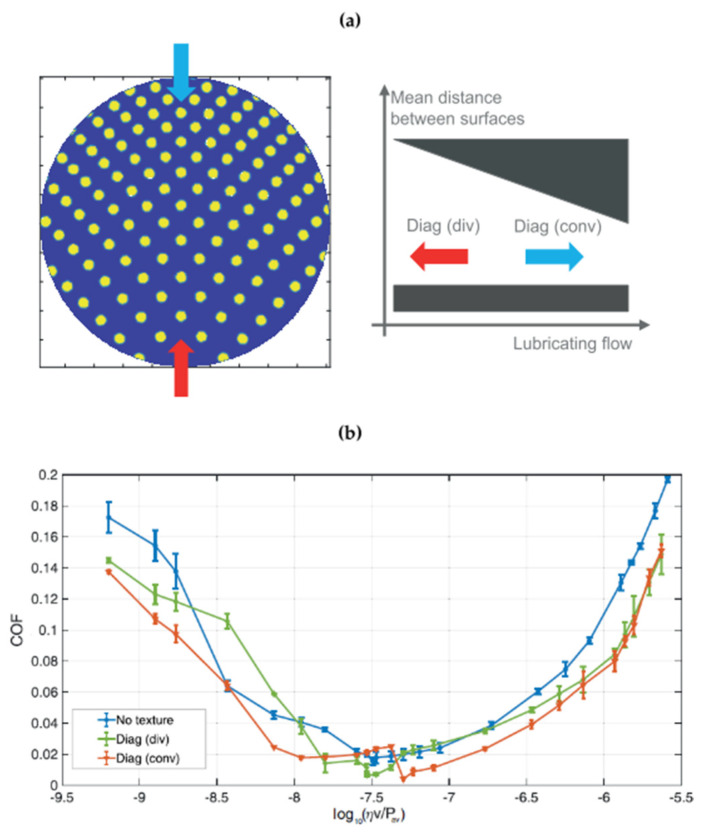
(**a**) Diagonal texture scheme (left) and equivalent convergent and divergent wedge (right); (**b**) COF vs. the Hersey number log10(ηv/Pav) for the Diagonal texture in convergent and divergent configurations. The load is constant 1 N. The untextured surface is shown for comparison. Reproduced from [34], under Creative Commons CC BY 4.0 license.

The results presented in Figure 23 were indeed in perfect agreement with the expectations based on the equivalent convergent and divergent wedge. In fact, inverting the flow direction, the differences between a convergent and a divergent wedge were highlighted, showing a general higher friction for the latter case. However, at very high velocities, micro/cavitation tended to prevail over collective effects, thus reducing the differences between the two configurations. Analogue results were also presented for the hydrostep pattern [34]. 

### 4.2. Non-Conformal Contacts

**Figure 24:**
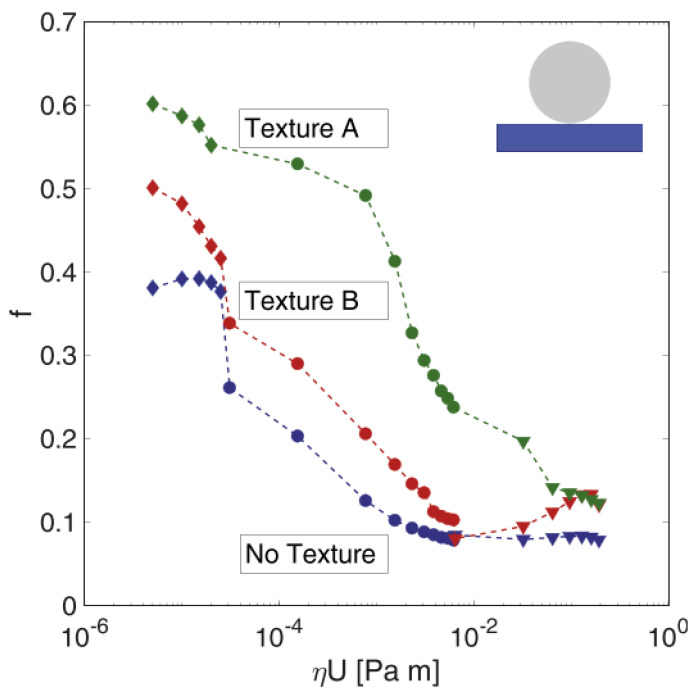
COF vs ηU in ball-on-disk tests. The normal load was of 1 N. Reproduced from [77], with permission from Elsevier.

Very different outcomes were obtained when testing non-conformal contact conditions, as shown in Figure 24.

Here, the authors did not find any improvement in terms of friction reduction with none of the textures but rather a sensitive worsening with texture A, over the whole Stribeck curve. In the mixed and elasto-hydrodynamic lubrication regimes, these results were ascribed to the low number of dimples involved in the contact area (few tens for Texture B and even lower for Texture A), which caused a reduced cavitation. In the boundary regime, where the role of dimples as micro-traps for wear particles was already established for hard materials [74], such a deterioration of the frictional characteristics was instead ascribed to the much bigger dimensions of the wear debris originating from long polymeric chains which can be hardly captured by the dimples [91]. Moreover, the authors also remarked that the lower wettability (compared to the untextured surface) induced by the laser treatment [92,93,94] hindered the formation of a lubricant film, which further resulted in larger values of the COF. 

**Figure 25:**
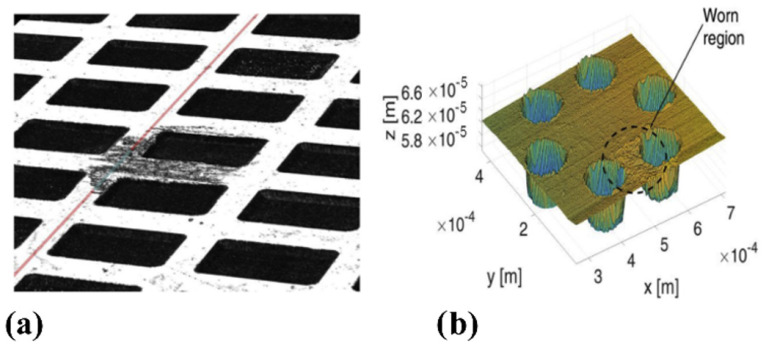
Worn region on (**a**) the rectangular dimples pattern and (**b**) the hexagonal texturing. Reproduced from [74], with permission from Elsevier.

Comparable outcomes were also found on stainless steel (1.4112) samples with two texture geometries consisting of circular dimples arranged on a hexagonal and a triangular lattice, tested in non-conformal contact conditions, where the contact area was comparable with the dimples size, as shown in Figure 25b [74]. The additional texture shown in Figure 25a was also fabricated and tested, consisting of denser (VR around 53%) rectangular dimples still with dimensions comparable with the contact area. 

**Figure 26:**
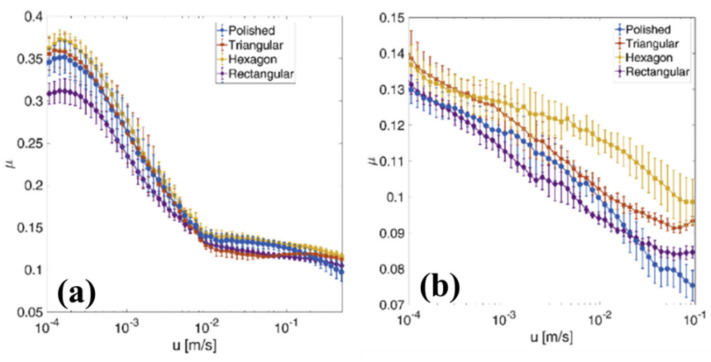
Friction coefficient vs. sliding velocity in case of (**a**): lubricant with a viscosity of 39.5 mm^2^/s at 20 °C and (**b**): lubricant with a viscosity of 1300 mm^2^/s at 40 °C. Reproduced from [74], with permission from Elsevier.

Here, the two different lubricants, with a viscosity around 39 mm^2^/s at 20 °C and 1300 mm^2^/s at 40 °C, respectively, were exploited for the tribological tests in order to highlight different lubrication regimes and to identify the most appropriate one to be used in mechanical apparatus. Figure 26 shows the measured values of the friction coefficient in the entire investigated sliding velocity range for the three uniform textured patterns with the polished sample used as a reference. In Figure 26a,b, the Stribeck curves obtained with the low- and high-viscosity lubricant are shown. With both lubricants, a significant deterioration of the frictional performances was noticed for the hexagonal and triangular patterns in comparison with the untextured. However, around 20% of friction reduction was achieved with the rectangular dimples textures [74]. 

The presence of the micro-texture was expected to increase wear compared to an untextured surface, due to a stress intensification mechanism, occurring during non-conformal contact because of the presence of sharp dimple edges in the contact region [95]. This was confirmed by the contact pressure measurements which revealed a significant increase in pressure at the location of the dimples and by the worn zone having approximately the same size of one dimple [74]. Higher contact pressures meant larger wear and also implicated more friction. However, such an effect cannot depend significantly on the dimple geometry. Therefore, the different frictional behavior of surfaces with different texture geometries was ascribed to another mechanism, i.e., to the entrapment of wear debris by the dimples. As already mentioned, this last effect is more relevant in the mixed and boundary lubrication regimes where the wear is larger [96]. The rectangular texture was characterized by a void ratio of 53%, which was significantly higher than the other two textures where the void ratios were equal to 27% (hexagonal) and 29% (triangular). A higher void ratio usually implies an increased capability to trap wear debris. Therefore, apparently this second mechanism ultimately prevailed over the stress intensification, leading to an overall friction reduction compared to the untextured. 

A review of recent results on laser surface texturing effect on the coefficient of friction is summarized in Table 1.

## 5. Conclusions

In the present review, laser surface texturing through direct laser writing (DLW) was described as a suitable technique to tune the friction properties of materials. In fact, it is widely recognized that modifying the surface topography of surfaces allows controlling their frictional properties. According to the specific conferred morphology it is possible to reduce the coefficient of friction but also increase it. Many mechanical applications would benefit from a reduction in the friction between mechanical mating surfaces, in order to prolong their life (by reducing the wear) and reduce the energy loss [97]. Nevertheless, in some cases, high friction is desirable, such as in tires or brakes. Controlling the friction by laser surface texturing is one of the most effective methods, due to its high degree of flexibility, originating by its CAD/CAM-based concept. Moreover, the use of ultrashort pulses prevents collateral damage which could result in local stresses at the dimples locations and then ensures high levels of accuracy and control of the micro-structures. Thanks to DLW a wide range of textures was reported in literature, characterized by diverse dimples’ shapes, depth, and spatial arrangements. This gave us the opportunity to explore the role of the micro-structures on the frictional properties, both as a local influence at the micro-structure sites and as a collective effect.

The experimental results regarding the frictional performances of the laser textured samples are presented in two contact regimes, i.e., conformal and non-conformal, to include the real working conditions which can be found in mechanical equipment, that is working operations involving the perfect match between the geometrical surfaces of the mating components or rather situations where the mating surfaces do not fit. The frictional properties were highlighted through the Stribeck curve, that is, the trend of the coefficient of friction as a function of a parameter which depends on the sliding speed between the mating surfaces, the lubricant viscosity, and the applied load. 

The reviewed results can be generalized as follows:

(1) Homogeneous arrangements of circular dimples allow a significant reduction the COF, the extent of the decrease being dependent on the geometry of the dimples (of the order of 20% with circular dimples with a diameter around 100 μm and an optimized depth around 6–7 μm; of the order of 80% with dimples with a diameter of 40 μm and depth of 4 μm. The specific arrangement of such micro-defects generated by direct laser writing over the sample surface revealed no to be significant especially in hydrodynamic lubrication regime, where cavitation played a more important role on the definition of the final tribological behavior.
(2) Directional textures of circular dimples (diameter of 180 μm and depth of 6.5 μm) highlighting the presence of equivalent convergent or divergent wedge, depending on the direction of the tests, reveal the possibility to dynamically switch between different frictional properties behavior, i.e., increase up to or reduction in the COF.
(3) Non-uniform textures consisting of elliptical or rectangular dimples (dimensions of 30 μm × 170 μm) having varying depth, give the possibility to obtain an important increase in the load capacity and the redirection of the lubricant fluid in the desired region of the surface.

Conversely, in non-conformal contact, the main role in the definition of the frictional properties is played by the number of dimples involved in the contact, i.e., by the void ratio. In fact, textures consisting of large dimples (diameter between 50 μm and 100 μm and depth between 10 μm and 50 μm) at a relatively low void ratio (between 20% and 35%) were detrimental for the tribological properties and led to a significant increase in the COF (up to 325%). Alternatively, increasing the void ratio up to 53% allowed us to overcome the stress intensification at the dimples edges and enhance the debris particle entrapment, thus leading to an overall friction reduction up to 20%.

## Figures and Tables

**Table 1:** Summary of the influence of laser surface textures on the coefficient of friction (COF).

Sample	Texture Design	Contact Type	Influence on COF	Reference
**Martensitic stainless steel X90CrMoV18 (1.4112)**	Crossed grooves	Conformal	Reduction Up to 33%	[47]
**Steel C85**	Hexagonal matrix circular dimples VR = 10%	Conformal	Reduction Up to 82%	[30]
**Steel C85**	Uniform grid Circular dimples VR = 10%	Conformal	Reduction Up to 82%	[30]
**100Cr6 steel**	Uniform grid Circular dimples VR = 33% and 44%	Conformal	Reduction Up to 60%	[34]
**Fluoroelastomer** **(commercial name Viton)**	Uniform grid Circular dimples VR = 35%	Non-conformal	Increase Up to 325%	[77]
**Fluoroelastomer** **(commercial name Viton)**	Uniform grid Circular dimples VR = 35%	Conformal	Reduction Up to 22%	[77]
**Fluoroelastomer** **(commercial name Viton)**	Uniform grid Circular dimples VR = 20%	Non-conformal	Increase Up to 28%	[77]
**Fluoroelastomer** **(commercial name Viton)**	Uniform grid Circular dimples VR = 20%	Conformal	Reduction Up to 60%	[77]
**Stainless steel (1.4112)**	Unifrom grid Rectangular dimples VR = 53%	Non-conformal	Reduction Up to 20%	[74]
**Chromium-hardened steel (AISI 52100) balls**	Uniform grid Circular dimples VR = 14%	Conformal	Reduction Up to 20%	[65]
**Steel C85**	Random matrix Circular dimples VR = 10%	Conformal	Reduction Up to 48%	[47]
**Chrome-coated steel**	Partial Texturing (symmetrical, at the ends of the sample) Circular dimples VR = 50%	Conformal	**Directional** Reduction Up to 29%	[38]
**100Cr6 steel**	Non -uniform design Five groups of inclined micro-grooved dimples with different depths	Conformal	**Directional** Reduction Up to 20%	[46]
**100Cr6 steel**	Fishbone design Circular dimples VR = 30%	Conformal	**Directional** Reduction Up to 55% Increase Up to 75%	[34]
**100Cr6 steel**	Diagonal matrix Circular dimples	Conformal	**Directional** Reduction Up to 25% Increase Up to 83%	[34]

## Data Availability

Not applicable.

## References

[1] Blau P.J. (2009). Friction Science and Technology From Concept to Applications.

[2] Marian M., Weikert T., Tremmel S. (2019). On Friction Reduction by Surface Modifications in the TEHL Cam/Tappet-Contact-Experimental and Numerical Studies. Coatings.

[3] Hamilton D.B., Walowit J.A., Allen C.M. (1966). A Theory of Lubrication by Micro- irregularities. Trans. ASME, J. Basic Eng..

[4] Anno J.N., Walowit J.A., Allen C.M. (1968). Microasperity Lubrication. J. Lubr. Technol..

[5] Pratap T., Patra K. (2018). Mechanical micro-texturing of Ti-6Al-4V surfaces for improved wettability and bio-tribological performances. Surf. Coat. Technol..

[6] Denkena B., Grove T., Schmidt C. (2018). Machining of Micro Dimples for Friction Reduction in Cylinder Liners. Procedia CIRP.

[7] Greco A., Raphaelson S., Ehmann K., Wang Q.J. (2016). Surface Texturing of Tribological Vibromechanical Texturing Method. J. Manuf. Sci. Eng. Trans..

[8] Wang X., Kato K., Adachi K., Aizawa K. (2003). Loads carrying capacity map for the surface texture design of SiC thrust bearing sliding in water. Tribol. Int..

[9] Etsion I. (2005). State of the art in laser surface texturing. J. Tribol..

[10] Gaudiuso C., Fanelli F., Mezzapesa F.P., Volpe A., Ancona A. (2023). Tailoring the wettability of surface-textured copper using sub-THz bursts of femtosecond laser pulses. Appl. Surf. Sci..

[11] Etsion I., Halperin G., Brizmer V., Kligerman Y. (2004). Experimental investigation of laser surface textured parallel thrust bearings. Tribol. Lett..

[12] Ronen A., Etsion I., Kligerman Y. (2001). Friction-reducing surface-texturing in reciprocating automotive components. Tribol. Trans..

[13] Chichkov B.N., Momma C., Nolte S., Von Alvensleben F., Tünnermann A. (1996). Femtosecond, picosecond and nanosecond laser ablation of solids. Appl. Phys. A Mater. Sci. Process..

[14] Gaudiuso C., Giannuzzi G., Volpe A., Lugarà P.M., Choquet I., Ancona A. (2018). Incubation during laser ablation with bursts of femtosecond pulses with picosecond delays. Opt. Express.

[15] Kerse C., Kalaycloĝ Lu H., Elahi P., Çetin B., Kesim D.K., Akçaalan Ö., Yavaş S., Aşlk M.D., Öktem B., Hoogland H. (2016). Ablation-cooled material removal with ultrafast bursts of pulses. Nature.

[16] Bonamis G., Audouard E., Hönninger C., Lopez J., Mishchik K., Mottay E., Manek-Hönninger I. (2020). Systematic study of laser ablation with GHz bursts of femtosecond pulses. Opt. Express.

[17] Mishchik K., Bonamis G., Qiao J., Lopez J., Audouard E., Mottay E., Hönninger C., Manek-Hönninger I. (2019). High-efficiency femtosecond ablation of silicon with GHz repetition rate laser source. Opt. Lett..

[18] Bauer F., Michalowski A., Kiedrowski T., Nolte S. (2015). Heat accumulation in ultra-short pulsed scanning laser ablation of metals. Opt. Express.

[19] Gaudiuso C., Terekhin P.N., Volpe A., Nolte S., Rethfeld B., Ancona A. (2021). Laser ablation of silicon with THz bursts of femtosecond pulses. Sci. Rep..

[20] Gaudiuso C., Stampone B., Trotta G., Volpe A., Ancona A. (2023). Investigation of the micro-milling process of steel with THz bursts of ultrashort laser pulses. Opt. Laser Technol..

[21] Hu J., Xu H. (2016). Friction and wear behavior analysis of the stainless steel surface fabricated by laser texturing underwater. Tribol. Int..

[22] Schille J., Schneider L., Mauersberger S., Szokup S., Höhn S., Pötschke J., Reiß F., Leidich E., Löschner U. (2020). High-Rate laser surface texturing for advanced tribological functionality. Lubricants.

[23] Cheng H., Wang W., Zhou Y., Qiao T., Lin W., Xu S., Yang Z. (2017). 5 GHz fundamental repetition rate, wavelength tunable, all-fiber passively mode-locked Yb-fiber laser. Opt. Express.

[24] (2013). Bharat Bhushan Introduction to Tribology.

[25] Hamrock B.J., Schmid S.R., Bo O., Marcel Dekker I. (2004). Jacobson Fundamental of Fluid Film Lubrication.

[26] Lu P., Wood R.J.K., Gee M.G., Wang L., Pfleging W. (2018). A Novel Surface Texture Shape for Directional Friction Control. Tribol. Lett..

[27] Lu P., Wood R.J.K., Gee M.G., Wang L., Pfleging W. (2017). The use of anisotropic texturing for control of directional friction. Tribol. Int..

[28] Scaraggi M., Mezzapesa F.P., Carbone G., Ancona A., Tricarico L. (2013). Friction Properties of Lubricated Laser-MicroTextured-Surfaces: An Experimental Study from Boundary- to Hydrodynamic- Lubrication. Tribol. Lett..

[29] Bhushan B., Handbook of Micro/Nano Tribology (1999). CRC Mechanics and Materials Science Series.

[30] Schneider J., Braun D., Greiner C. (2017). Laser textured surfaces for mixed lubrication: Influence of aspect ratio, textured area and dimple arrangement. Lubricants.

[31] Hsu S.M., Jing Y., Hua D., Zhang H. (2014). Friction reduction using discrete surface textures: Principle and design. J. Phys. D. Appl. Phys..

[32] Yu H., Wang X., Zhou F. (2010). Geometric Shape Effects of Surface Texture on the Generation of Hydrodynamic Pressure Between Conformal Contacting Surfaces. Tribol. Lett..

[33] Qiu M., Delic A., Raeymaekers B. (2012). The Effect of Texture Shape on the Load-Carrying Capacity of Gas-Lubricated Parallel Slider Bearings. Tribol. Lett..

[34] Putignano C., Parente G., Profito F.J., Gaudiuso C., Ancona A., Carbone G. (2020). Laser Microtextured Surfaces for Friction Reduction: Does the Pattern Matter?. Materials.

[35] Zhong Y., Zheng L., Gao Y., Liu Z. (2019). Numerical simulation and experimental investigation of tribological performance on bionic hexagonal textured surface. Tribol. Int..

[36] Scaraggi M. (2015). Partial surface texturing: A mechanism for local flow reconditioning in lubricated contacts. Proc. Inst. Mech. Eng. Part J. Eng. Tribol..

[37] Ancona A., Carbone G., Filippis M.D., Volpe A., Lugarà P.M. (2014). Femtosecond laser full and partial texturing of steel surfaces to reduce friction in lubricated contact. Adv. Opt. Technol..

[38] Ryk G., Kligerman Y., Etsion I., Shinkarenko A. (2005). Experimental investigation of partial laser surface texturing for piston-ring friction reduction. Tribol. Trans..

[39] Etsion I. (2004). Improving tribological performance of mechanical components by laser surface texturing. Tribol. Lett..

[40] Scaraggi M., Mezzapesa F.P., Carbone G., Ancona A., Sorgente D., Lugarà P.M. (2014). Minimize friction of lubricated laser-microtextured-surfaces by tuning microholes depth. Tribol. Int..

[41] Etsion I., Bruce R.W. (2012). Handbook of Lubrication and Tribology, Volume II: Theory and Design.

[42] Brizmer V., Kligerman Y., Etsion I. (2003). A laser surface textured parallel thrust bearing. Tribol. Trans..

[43] Scaraggi M. (2014). Optimal Textures for Increasing the Load Support in a Thrust Bearing Pad Geometry. Tribol. Lett..

[44] Costa H.L., Schille J., Rosenkranz A. (2022). Tailored surface textures to increase friction—A review. Friction.

[45] Scaraggi M. (2012). Textured surface hydrodynamic lubrication: Discussion. Tribol. Lett..

[46] Ancona A., Joshi G.S., Volpe A., Scaraggi M., Lugarà P.M., Carbone G. (2017). Non-uniform laser surface texturing of an un-tapered square pad for tribological applications. Lubricants.

[47] Stark T., Alamri S., Aguilar-Morales A.I., Kiedrowski T., Lasagni A.F. (2019). Positive effect of laser structured surfaces on tribological performance. J. Laser Micro Nanoeng..

[48] Stark T., Kiedrowski T., Marschall H., Lasagni A.F. (2019). Avoiding starvation in tribocontact through active lubricant transport in laser textured surfaces. Lubricants.

[49] Nolte S., Momma C., Jacobs H., Tünnermann A., Chickov B.N., Wellegehausen B., Welling H. (1997). Ablation of metals by ultrashort laser pulses. J. Opt. Soc. Am. B.

[50] Moradi S., Kamal S., Englezos P., Hatzikiriakos S.G. (2013). Femtosecond laser irradiation of metallic surfaces: Effects of laser parameters on superhydrophobicity. Nanotechnology.

[51] Romano J., Garcia-giron A., Penchev P., Dimov S. (2018). Triangular laser-induced submicron textures for functionalising stainless steel surfaces. Appl. Surf. Sci..

[52] Belloni V.V., Bollani M., Eaton S.M., Di Trapani P., Jedrkiewicz O. (2021). Micro-hole generation by high-energy pulsed bessel beams in different transparent materials. Micromachines.

[53] Gaudiuso C., Volpe A., Ancona A. (2020). One-Step Femtosecond Laser Stealth Dicing of Quartz. Micromachines.

[54] Lasagni A.F. (2017). Laser interference patterning methods: Possibilities for high-throughput fabrication of periodic surface patterns. Adv. Opt. Technol..

[55] Aguilar-Morales A.I., Alamri S., Lasagni A.F. (2018). Micro-fabrication of high aspect ratio periodic structures on stainless steel by picosecond direct laser interference patterning. J. Mater. Process. Technol..

[56] Bieda M., Siebold M., Lasagni A.F. (2016). Fabrication of sub-micron surface structures on copper, stainless steel and titanium using picosecond laser interference patterning. Appl. Surf. Sci..

[57] Lasagni A., Benke D., Kunze T., Bieda M., Eckhardt S., Roch T., Langheinrich D., Berger J. (2015). Bringing the direct laser interference patterning method to industry: A one tool-complete solution for surface functionalization. J. Laser Micro Nanoeng..

[58] Mezera M., Florian C., Römer G.W., Krüger J., Bonse J. (2023). Creation of Material Functions by Nanostructuring.

[59] Bonse J., Hohm S., Kirner S.V., Rosenfeld A., Kruger J. (2017). Laser-Induced Periodic Surface Structures— A Scientific Evergreen. IEEE J. Sel. Top. Quantum Electron..

[60] Giannuzzi G., Gaudiuso C., Franco C.D., Scamarcio G., Lugarà P.M., Ancona A. (2019). Large area laser-induced periodic surface structures on steel by bursts of femtosecond pulses with picosecond delays. Opt. Lasers Eng..

[61] Giannuzzi G., Gaudiuso C., Di Mundo R., Mirenghi L., Fraggelakis F., Kling R., Lugarà P.M., Ancona A. (2019). Short and long term surface chemistry and wetting behaviour of stainless steel with 1D and 2D periodic structures induced by bursts of femtosecond laser pulses. Appl. Surf. Sci..

[62] Bonse J., Gräf S. (2021). Ten open questions about laser-induced periodic surface structures. Nanomaterials.

[63] Römer G.R.B.E., Skolski J.Z.P., Oboňa J.V., Ocelík V., de Hosson J.T.M. (2014). Laser-induced periodic surface structures, modeling, experiments, and applications. Laser-Based Micro-Nanoprocessing VIII.

[64] Mao B., Siddaiah A., Liao Y., Menezes P.L. (2020). Laser surface texturing and related techniques for enhancing tribological performance of engineering materials: A review. J. Manuf. Process..

[65] Boidi G., Grützmacher P.G., Kadiric A., Profito F.J., Machado I.F., Gachot C., Dini D. (2021). Fast laser surface texturing of spherical samples to improve the frictional performance of elasto-hydrodynamic lubricated contacts. Friction.

[66] Mannion P.T., Magee J., Coyne E., O’Connor G.M., Glynn T.J. (2004). The effect of damage accumulation behaviour on ablation thresholds and damage morphology in ultrafast laser micro-machining of common metals in air. Appl. Surf. Sci..

[67] Di Niso F., Gaudiuso C., Sibillano T., Mezzapesa F.P., Ancona A., Lugarà P.M. (2014). Role of heat accumulation on the incubation effect in multi-shot laser ablation of stainless steel at high repetition rates. Opt. Express.

[68] Gropper D., Wang L., Harvey T.J. (2016). Hydrodynamic lubrication of textured surfaces: A review of modeling techniques and key findings. Tribol. Int..

[69] Gachot C., Rosenkranz A., Hsu S.M., Costa H.L. (2017). A critical assessment of surface texturing for friction and wear improvement. Wear.

[70] Sudeep U., Tandon N., Pandey R.K. (2015). Performance of Lubricated Rolling/Sliding Concentrated Contacts With Surface Textures: A Review. J. Tribol..

[71] Rosenkranz A., Grützmacher P.G., Gachot C., Costa H.L. (2019). Surface Texturing in Machine Elements − A Critical Discussion for Rolling and Sliding Contacts. Adv. Eng. Mater..

[72] Grützmacher P.G., Profito F.J., Rosenkranz A. (2019). Multi-scale surface texturing in tribology-current knowledge and future perspectives. Lubricants.

[73] Wang L., Zhao X., He M., Zhang W. (2021). Effect of micro grooves on lubrication performance of friction pairs. Meccanica.

[74] Joshi G.S., Putignano C., Gaudiuso C., Stark T., Kiedrowski T., Ancona A., Carbone G. (2018). Effects of the micro surface texturing in lubricated non-conformal point contacts. Tribol. Int..

[75] Li X., Li Y., Tong Z., Ma Q., Ni Y., Dong G. (2019). Enhanced lubrication effect of gallium-based liquid metal with laser textured surface. Tribol. Int..

[76] Yan S., Wei C., Zou H., Chen J., Li Y., Shen T., Wang A., Sui T., Lin B. (2021). Fabrication and tribological characterization of laser textured engineering ceramics: Si3N4, SiC and ZrO2. Ceram. Int..

[77] Putignano C., Scarati D., Gaudiuso C., Di Mundo R., Ancona A., Carbone G. (2019). Soft matter laser micro-texturing for friction reduction: An experimental investigation. Tribol. Int..

[78] Rosenkranz A., Szurdak A., Grützmacher P.G., Hirt G., Mücklich F. (2018). Friction Reduction Induced by Elliptical Surface Patterns under Lubricated Conditions. Adv. Eng. Mater..

[79] Wang J., Wang Q., Li Y., Guo M., Li P., Li Y. (2021). Numerical and experimental study on the effect of surface texture with roughness orientation considered under a mixed lubrication state. Ind. Lubr. Tribol..

[80] Shinkarenko A., Kligerman Y., Etsion I. (2009). The effect of elastomer surface texturing in soft elasto-hydrodynamic lubrication. Tribol. Lett..

[81] Ramesh A., Akram W., Mishra S.P., Cannon A.H., Polycarpou A.A., King W.P. (2013). Friction characteristics of microtextured surfaces under mixed and hydrodynamic lubrication. Tribol. Int..

[82] Peng X.D., Sheng S.E., Li J.Y., Pan X.M., Bai S.X. (2008). Effects of dimple geometric parameters on the performance of a laser-textured mechanical seal. Key Eng. Mater..

[83] Braun D., Greiner C., Schneider J., Gumbsch P. (2014). Efficiency of laser surface texturing in the reduction of friction under mixed lubrication. Tribol. Int..

[84] Greiner C., Merz T., Braun D., Codrignani A., Magagnato F. (2015). Optimum dimple diameter for friction reduction with laser surface texturing: The effect of velocity gradient. Surf. Topogr. Metrol. Prop..

[85] Yan D., Qu N., Li H., Wang X. (2010). Significance of dimple parameters on the friction of sliding surfaces investigated by orthogonal experiments. Tribol. Trans..

[86] Kovalchenko A., Ajayi O., Erdemir A., Fenske G., Etsion I. (2005). The effect of laser surface texturing on transitions in lubrication regimes during unidirectional sliding contact. Tribol. Int..

[87] Vlădescu S., Medina S., Olver A.V., Pegg I.G., Reddyhoff T. (2016). The Transient Friction Response of a Laser-Textured, Reciprocating Contact to the Entrainment of Individual Pockets. Tribol. Lett..

[88] Segu D.Z., Choi S.G., Choi J.H., Kim S.S. (2013). The effect of multi-scale laser textured surface on lubrication regime. Appl. Surf. Sci..

[89] Rosenkranz A., Costa H.L., Profito F., Gachot C., Medina S., Dini D. (2019). Influence of surface texturing on hydrodynamic friction in plane converging bearings—An experimental and numerical approach. Tribol. Int..

[90] Wos S., Koszela W., Dzierwa A., Pawlus P. (2020). Friction reduction in unidirectional lubricated sliding due to disc surface texturing. Coatings.

[91] Persson B.N.J. (2009). Theory of powdery rubber wear. J. Phys. Condens. Matter.

[92] Gaudiuso C. (2023). Applied Sciences Laser Fabrication: A Solid Present for the Brightest Future. Appl. Sci..

[93] Lawrence J., Waugh D.G. (2017). Creating superhydrophobic surface structures via the rose petal effect on stainless steel with a picosecond laser. Lasers Eng..

[94] Cardoso J.T., Aguilar-Morales A.I., Alamri S., Huerta-Murillo D., Cordovilla F., Lasagni A.F., Ocaña J.L. (2018). Superhydrophobicity on hierarchical periodic surface structures fabricated via direct laser writing and direct laser interference patterning on an aluminium alloy. Opt. Lasers Eng..

[95] Sackfield A., Dini D., Hills D.A. (2007). Contact of a rotating wheel with a flat. Int. J. Solids Struct..

[96] Vlădescu S., Ciniero A., Tufail K., Reddyhoff T. (2018). Optimization of Pocket Geometry for Friction Reduction in Piston—Liner Contacts. Tribol. Trans..

[97] Nakashima K., Matsunaga K., Uchiyama Y., Yoshida M. (2023). Development of measurement apparatus of piston assembly friction in a small motorcycle engine. Combust. Engines.

